# Neurobiological and neuropsychological disturbance in EDS

**DOI:** 10.3389/fneur.2025.1648702

**Published:** 2025-10-31

**Authors:** Matt Westerman, Alex Kafkas, Adrian Parry-Jones, Samantha Strong, Chris Retzler, Glyn Hallam

**Affiliations:** ^1^Faculty of Biology, Medicine and Health, School of Health Sciences, University of Manchester, Manchester, United Kingdom; ^2^Geoffrey Jefferson Brain Research Centre, Manchester Academic Health Science Centre, Northern Care Alliance, University of Manchester, Manchester, United Kingdom; ^3^Division of Cardiovascular Sciences, Faculty of Biology, Medicine and Health, University of Manchester, Manchester, United Kingdom; ^4^College of Health and Life Sciences, Aston University, Birmingham, United Kingdom; ^5^Department of Social and Psychological Sciences, University of Huddersfield, Huddersfield, United Kingdom; ^6^School of Education, Language and Psychology, York St John University, York, United Kingdom

**Keywords:** Ehlers Danlos syndrome, emotional dysregulation, anxiety, chronic pain, Chiari malformation, cerebrovascular, interoception

## Abstract

Ehlers-Danlos Syndrome (EDS) is a collection of connective tissue disorders, defined by genetic defects in collagen and extracellular matrix proteins that lead to joint hypermobility, skin fragility, and vascular complications. However, recent studies point to a broader impact, revealing how EDS has both neurological and psychological effects. This review explores these neurological and neuropsychological dimensions of EDS across its 13 subtypes, drawing together evidence on brain structure changes such as Chiari malformations and craniocervical instability, alongside small fibre neuropathy, blood–brain barrier vulnerabilities, and cerebrovascular risks, particularly prevalent in the vascular EDS subtype. The review will also explore how these physical disruptions may act upon mental health, fueling anxiety, mood instability, and cognitive challenges. Mechanisms such as neuroinflammation, altered interoception, and chronic pain may contribute to these effects and drive emotional dysregulation. By reviewing clinical observations, neuroimaging findings, and emerging theories, this paper highlights the importance of understanding the involvement of the brain in EDS. The review highlights the need for a shift in approach to EDS, and an integrated effort across neurology, psychiatry, and genetics to better support those living EDS.

## 1 Introduction

Ehlers-Danlos Syndromes (EDS) represent a heterogeneous group of 13 heritable connective tissue disorders, unified by genetic mutations that disrupt collagen synthesis, processing, or extracellular matrix (ECM) stability (1). The complex clinical presentation, diagnostic challenges, symptom overlap with other disorders (e.g., fibromyalgia, chronic fatigue syndrome, and anxiety disorders), and limited medical education on the topic, has led to several misunderstandings and misconceptions about the condition (2). Historically, EDS has been characterised by musculoskeletal problems, such as recurrent joint dislocations, hypermobility, hyperextensibility, soft tissue injuries, and dermatological features increased elasticity and fragility of skin. However, evidence suggests that EDS extends far beyond these physical domains, impacting neurological and neuropsychological functions. Such impacts include alterations in brain structure, autonomic nervous system regulation, pain processing, sensory integration, and cognitive performance, often with significant implications for patients’ quality of life (3, 4). This review aims to explore these understudied aspects, bridging the gap between connective tissue pathology and neurological complications. We aim to provide a foundation for advancing clinical management and research priorities in this complex syndrome.

It is important to distinguish between Joint Hypermobility Syndrome (JHS), Hypermobility Spectrum Disorders (HSD), and hypermobile Ehlers–Danlos Syndrome (hEDS). Historically, JHS was often confused with hEDS under diagnostic frameworks like the Brighton or Villefranche criteria, leading to inconsistent prevalence estimates. The 2017 International Classification introduced stricter criteria for hEDS, while those with symptomatic hypermobility not meeting full hEDS thresholds are now classified under HSD. In this review, we use “hEDS” to refer to cases meeting the 2017 criteria and reserve “JHS” only for studies predating 2017 to maintain precision and consistency with current diagnostic frameworks. The 2017 classification system refined the diagnostic criteria for hEDS, introducing stricter thresholds for joint hypermobility and additional systemic features to distinguish it from JHS and other connective tissue disorders (1). In this review, “hEDS” is used in line with these updated criteria, with references to JHS reserved for studies predating 2017 to ensure terminological consistency.

## 2 Genetic mutations of Ehlers Danlos syndrome

**Figure 1:**
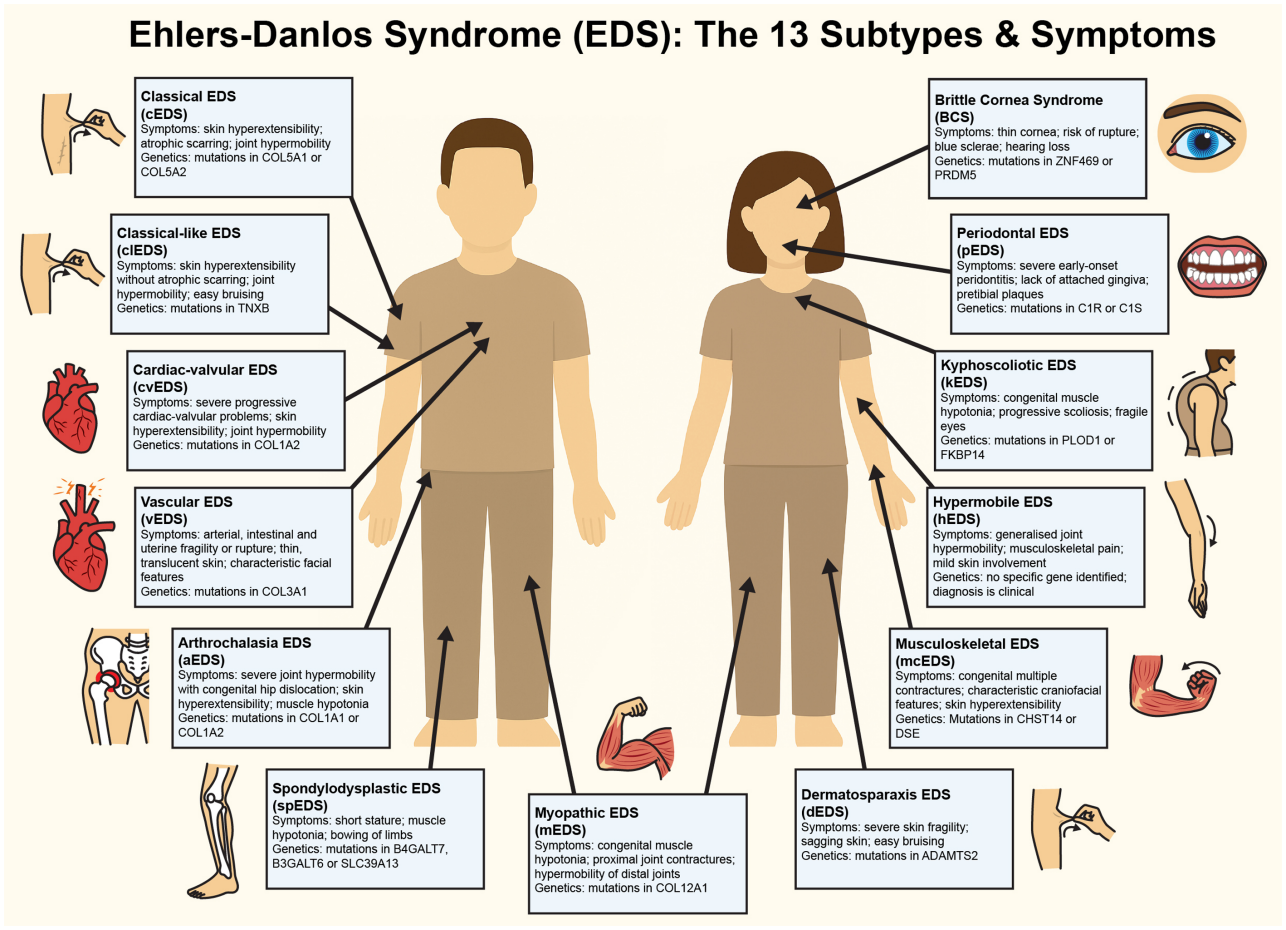
Figure detailing the symptoms of each of the known 13 subtypes of Ehlers-Danlos Syndrome. This diagram illustrates the clinical features and genetic mutations characteristic of each EDS subtype. Illustrations map the clinical symptoms to anatomical locations for visual clarity.

The 13 EDS subtypes, as defined by the 2017 International Classification, each present unique genetic etiologies and clinical profiles (see Figure 1). Classical EDS (cEDS) is driven by mutations in COL5A1 or COL5A2, and manifests as highly elastic yet fragile skin prone to tearing, atrophic scarring from poor wound healing, and joint hypermobility that predisposes individuals to frequent dislocations and chronic pain (5). Hypermobile EDS (hEDS) is the most prevalent subtype, and it is characterised by generalised joint hypermobility, widespread musculoskeletal pain, gastrointestinal dysmotility (e.g., gastroparesis), and autonomic dysfunction such as postural orthostatic tachycardia syndrome (POTS). Despite its widespread occurrence within the population, the genetic basis of hEDS’s remains elusive, with rare cases linked to TNXB gene mutations and ongoing research exploring polygenic contributions (6). Vascular EDS (vEDS) is often considered the most serious form of EDS, and is caused by COL3A1 mutations that result in the life-threatening fragility of blood vessels and hollow organs, leading to arterial dissections, spontaneous organ rupture, easy bruising, and translucent skin with distinctive facial features (1). Kyphoscoliotic EDS (kEDS), associated with PLOD1 or FKBP14 gene mutations, presents with severe congenital hypotonia, progressive kyphoscoliosis, ocular fragility (e.g., scleral rupture), joint contractures, and, in some PLOD1 cases, hearing loss (7).

Less common subtypes further underscore the impact of EDS on the lives of EDS patients. Dermatosparaxis EDS (dEDS), resulting from ADAMTS2 gene mutations, leads to severely fragile, sagging skin and delayed wound healing, with potential proprioceptive deficits from tissue laxity (1). Brittle cornea syndrome (BCS), tied to ZNF469 or PRDM5 gene defects, compromises ocular integrity, thus increasing risks of corneal rupture and visual impairment that may subtly affect spatial cognition (8). Arthrochalasia EDS (aEDS), caused by COL1A1 or COL1A2 genetic mutations, has symptoms of extreme joint laxity, recurrent dislocations, and congenital hip dysplasia, often resulting in proprioceptive imprecision that impairs coordination and elevates injury risk. This can potentially contribute to psychological distress through chronic pain and reduced mobility (1). Musculocontractural EDS (mcEDS; CHST14 or DSE) includes adducted thumbs, clubfoot, craniofacial anomalies, and intellectual disability, suggesting neurodevelopmental involvement (9). Spondylodysplastic EDS (spEDS; B4GALT7, B3GALT6, or SLC39A13) presents clinically with short physical stature, muscle hypotonia, and skeletal dysplasia, while myopathic EDS (mEDS; COL12A1 or TPM2) involves progressive muscle atrophy and weakness, both potentially intersecting with neurological deficits (10–12).

## 3 Prevelance of Ehlers Danlos syndrome

The estimated prevalence of EDS across the subtypes has varied across studies. Early research (13) suggested that EDS affects approximately 1 in 5,000 individuals. This figure is supported by population data from Denmark, where (14) reported a similar population prevalence of 0.02%. However, more recent data from a Welsh healthcare records study (15) indicated a higher prevalence of around 1 in 500 (0.2% prevalence). These findings suggest that earlier estimates may underestimate the true prevalence, particularly for hEDS, which, even as the most common subtype, is thought to be more common than previously recognised. This highlights that further large-scale population studies are required to confirm an accurate prevalence of EDS, particularly as historically, joint hypermobility syndrome (JHS) was often conflated with hEDS under diagnostic frameworks such as the Brighton and Villefranche criteria (16), leading to overlap in early research findings. Regarding other EDS subtypes, prevalence figures indicate that they are significantly rarer. Classical EDS is estimated to occur in approximately 1 in 20,000 to 40,000 individuals, while vascular EDS affects about 1 in 100,000 to 200,000 people (2). The remaining subtypes are classified as ultra-rare, affecting fewer than 1 in a million individuals (1). Furthermore, aEDS is an exceptionally rare subtype of EDS of which approximately 42 cases have been documented worldwide (17). Again, these figures highlight the diverse nature of EDS and the need for ongoing research to better understand the prevalence and clinical impact across the population.

By exploring the neurological and neuropsychological dimensions of EDS, including cognitive dysfunction, autonomic dysregulation, and heightened anxiety, this review aims to reconceptualise the condition as a mind–body disorder. Such an approach advocates for integrated care models that simultaneously address the physical, neurological, and psychological challenges faced by individuals with EDS, moving beyond traditional musculoskeletal-focused management strategies. Beyond connective tissue pathology, the musculoskeletal system plays a critical role in shaping psychological well-being in EDS. Chronic joint instability, recurrent dislocations, and reduced muscle tone can impair proprioception and motor unit efficiency, contributing to fatigue and physical vulnerability. These disruptions may heighten bodily vigilance and undermine confidence in movement, thereby perpetuating anxiety and emotional dysregulation. Linking musculoskeletal deficits with neuropsychological outcomes emphasises the need for integrated models that consider both physical and mental health trajectories in EDS.

## 4 Neurological implications of EDS—structural and functional brain changes

EDS is becoming increasingly recognised for its neurological abnormalities, with patients presenting with structural brain changes, functional impairments, and sensory processing deficits that challenge the traditional view of the syndrome as only musculoskeletal. Chiari malformation type I (CM-I), prevalent in hEDS, involves cerebellar tonsil displacement into the spinal canal, disrupting CSF flow and causing occipital headaches, vertigo, nystagmus, and coordination difficulties (3). This anomaly may compress brainstem structures, such as the medulla oblongata and upper cervical spinal cord, potentially leading to a range of neurological symptoms, including dysphagia (difficulty swallowing), sleep apnea, tinnitus, balance problems, and even syncope, potentially affecting respiratory control and autonomic stability (18). However, it remains unclear to what extent cerebellar abnormalities affect attention (19) or how prefrontal-CSF interactions might impair executive function, both of which are critical for everyday cognitive performance. Craniocervical instability (CCI) is also a frequent clinical presentation in hEDS, and is caused by lax ligaments at the skull-cervical junction, leading to neck pain, muscle weakness, and cranial nerve dysfunction (e.g., dysphagia, tinnitus). CCI’s mechanical stress on the brainstem and upper spinal cord may contribute to dysautonomia (e.g., postural orthostatic tachycardia syndrome (POTS)) and subtle cognitive deficits, possibly via disrupted prefrontal connectivity, however, longitudinal studies on such brain changes are lacking so it is difficult to fully appreciate the extent of these effects (4).

Indeed, recent research has shown that EDS patients are more prone to suffer from headaches (75% of the EDS population suffer with recurrent headaches), than the general population (20). This study also highlights the occurrence of orthostatic headaches, which may indicate underlying conditions such as spinal cerebrospinal fluid leaks, dysautonomia, or craniocervical abnormalities, all of which have been associated with heritable connective tissue disorders (20). Another recent study by Wu and Ho (21) highlights the frequent co-occurrence of POTS and gastrointestinal disturbances, such as gastroparesis, in EDS patients. POTS is characterised by orthostatic intolerance and tachycardia which reflects underlying disruptions in autonomic nervous system regulation and is potentially linked to brainstem and cerebellar processing deficits (22). POTS may also be thought of as a central nervous system disorder (22) as symptoms are known to contribute to cognitive dysfunction, including impairments in attention and memory, potentially mediated by transient cerebral hypoperfusion. Moreover, chronic gastrointestinal symptoms may exacerbate emotional dysregulation, likely through altered interoceptive signalling along the gut-brain axis (see section 5 for a review on interoception). These findings suggest that EDS may elicit and contribute to neurological and psychological factors, impacting the individual’s quality of life through central nervous system dysregulation.

In a similar fashion, small fibre neuropathy (SFN) emerges as a unifying neurological feature across subtypes like hEDS, vEDS, and cEDS, characterised by degeneration of small unmyelinated nerve fibres. Confirmed by reduced intraepidermal nerve fibre density on skin biopsies, SFN drives chronic neuropathic pain, sensory disturbances (e.g., burning, tingling), and autonomic symptoms like gastrointestinal dysmotility and orthostatic intolerance (23). Its presentation suggests that it is associated with a peripheral nerve vulnerability tied to ECM defects, potentially exacerbating central pain processing abnormalities. Neurodevelopmental anomalies further complicate the picture; periventricular heterotopia (clusters of misplaced neurons from faulty migration) occur in some EDS cases, and are associated with epilepsy, developmental delays, and cognitive impairment (24). These findings hint at a broader developmental role for collagen in neural organisation, warranting genetic and histological investigation.

Connective tissue fragility in EDS may also heighten susceptibility to traumatic brain injury (plausibly linked to structural white matter weakness within the meningeal and perivascular connective tissues) with even mild impacts resulting in neurological symptoms like headaches, dizziness, or memory lapses (25, 26). In hEDS, proprioceptive difficulties from joint laxity are hypothesised to mediate links between hypermobility, neurodivergence (e.g., autism, ADHD), and emotional dysregulation, possibly via altered cerebellar-thalamic circuits however, this remains a preliminary model awaiting neuroimaging support (27). Periodontal EDS (pEDS), reveals white matter abnormalities and small vessel disease on MRI, with leukoencephalopathy suggesting microvascular fragility. While some patients exhibit minimal neurological symptoms, others report cognitive decline, ataxia, or seizures, indicating variable penetrance that demands larger cohort studies (28). Neuroimaging in individuals with joint hypermobility shows reduced grey matter volume in midline cortical regions such as the anterior cingulate cortex, which is heavily implicated in pain and emotional regulation, suggesting subtype-specific brain alterations that may generalise to other forms (29). Furthermore, in anxious patients with JHS/hEDS, insular volume was larger and linked to peak heart rate on standing, while amygdala was volume related to both hypermobility and interoceptive accuracy (30). These findings elucidate the need for subtype-stratified MRI studies to understand the neurological and neuropsychological aspects of EDS.

## 5 Blood–brain barrier dysfunction and neuroinflammation

**Figure 2:**
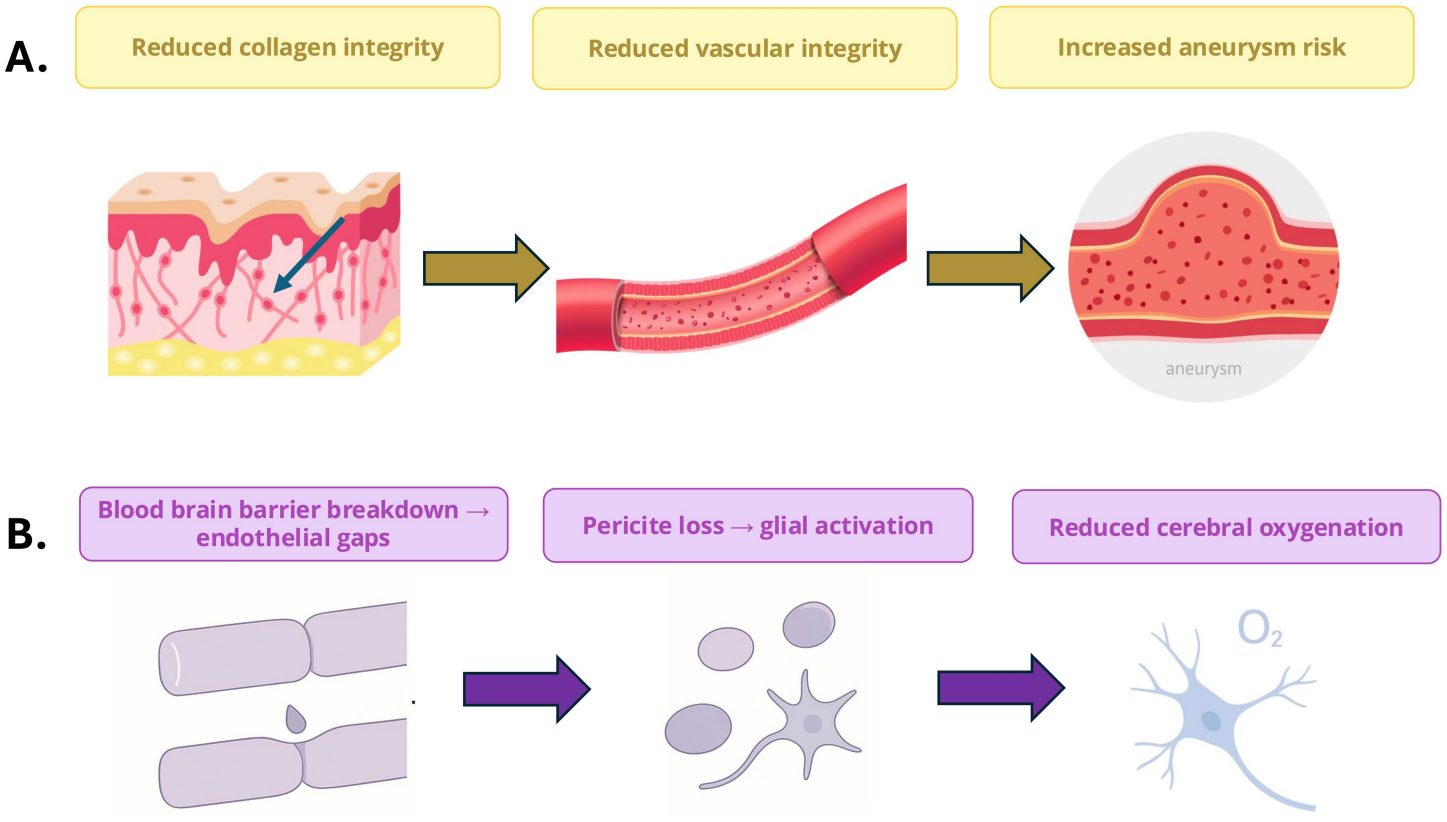
Connective tissue and neurovascular dysfunction in Ehlers–Danlos Syndrome (EDS). **(A)** Defective collagen organisation reduces vascular integrity and increases susceptibility to aneurysm formation. **(B)** Blood–brain barrier disruption and endothelial gaps promote pericyte loss, glial activation, and reduced cerebral oxygenation, forming a mechanistic link between connective tissue fragility and neurovascular dysfunction.

The blood–brain barrier (BBB) maintains the brain’s homeostasis by regulating the passage of substances from the bloodstream into the central nervous system (CNS). In Ehlers-Danlos Syndrome (EDS), collagen and ECM defects in EDS may compromise the BBB potentially increasing its permeability and predisposing patients to neuroinflammation (31). In vEDS, it is known that defective type 3 collagen (COL3A1) weakens vascular endothelium and perivascular ECM, heightening risks of cerebrovascular events (e.g., arterial dissections) and possibly allowing pro-inflammatory cytokines (e.g., IL-6, TNF-*α*) to breach the CNS (see Figure 2). In hEDS and general hypermobile EDS subtypes, connective tissue laxity contributes to structural anomalies like Chiari and CCI, which disrupt CSF dynamics which can impact intracranial pressure resulting in inflammation (32). While direct evidence of BBB dysfunction in EDS (e.g., CSF cytokine elevations, gadolinium leakage) is absent, the hypothesis aligns with known collagen roles in vascular integrity and offers a plausible mechanism for some neurological symptoms.

Neuroinflammation may drive a cascade of CNS effects in EDS. Heightened amygdala reactivity under inflammatory conditions could amplify emotional responses, while prefrontal cortex hypoactivity impairs top-down regulation, leading to mood instability and anxiety (33, 34). Hippocampal inflammation, which has been linked to depressive states (35), might exacerbate emotional dysregulation and compound EDS’s psychological burden. Given the hippocampus’s central role in episodic memory, such inflammation could also contribute to broader cognitive difficulties. Other aspects may further impact this, such as CSF pressure changes from Chiari or CCI disrupted sleep patterns (irritability and cognitive fog), both prominent in EDS cohorts. Chronic pain and fatigue, ubiquitous in all forms of EDS, may further impact inflammatory pathways, creating a feedback loop with emotional and neurological decline (36). Intriguingly, anecdotal reports suggest a higher incidence of demyelinating diseases like multiple sclerosis in EDS, potentially tied to BBB vulnerability or shared ECM dysregulation however such association should be taken with caution as epidemiological data are lacking to substantiate this claim (37).

The interplay between BBB dysfunction, neuroinflammation, and structural anomalies likely underpins a spectrum of EDS symptoms, from sensory disturbances to psychiatric sequelae. For example, in vEDS, BBB breaches could combine with vascular fragility to heighten stroke-independent CNS inflammation, while in hEDS, CSF-driven inflammation might link dysautonomia to cognitive complaints. Validation requires direct measures such as CSF cytokine profiling, BBB permeability assays (e.g., DCE-MRI), or post-mortem histology, positioning this as a frontier for EDS neuroscience research.

## 6 Musculoskeletal contributions to psychological well-being in Ehlers–Danlos syndrome

Musculoskeletal manifestations in Ehlers–Danlos Syndrome (EDS) are not merely physical burdens but play a central role in shaping the emotional and psychological well-being of affected individuals. Chronic musculoskeletal pain is one of the most prevalent symptoms across EDS subtypes and is strongly associated with anxiety, depressive disorders, and reduced quality of life (3, 38). Patients frequently describe unrelenting joint and muscle pain as both physically disabling and emotionally exhausting, with large cohort studies showing significantly higher rates of depression and anxiety among EDS populations compared to the general public (39, 40). Pain often fosters catastrophising and fear of movement, creating a feedback loop in which anticipation of pain heightens stress reactivity and reduces engagement in daily activities (41). This cycle is compounded by activity limitations and social withdrawal, which can erode self-esteem and contribute to feelings of isolation (42). Furthermore, visible musculoskeletal consequences, such as scarring in classical EDS (cEDS) or the need for mobility aids in severe hypermobile EDS (hEDS), may expose patients to stigma or bullying, particularly in childhood and adolescence, further amplifying psychological distress (36). Even beyond pain, proprioceptive dysfunction has emerged as a distinctive musculoskeletal-neurological bridge to psychological outcomes. Damage to joint mechanoreceptors and connective tissue laxity contribute to imprecise body-position signals, leaving patients with clumsiness, balance deficits, and heightened injury risk (27, 43). This sensory unreliability often manifests as kinesiophobia, defined as an excessive fear of movement, since patients experience a constant anticipation of pain or harm (44, 45). In particular, this has been related not to the intensity of pain, rather the association between musculoskeletal pain and fatigue (43).

Alongside proprioceptive and pain-related mechanisms, muscle weakness, motor dysfunction, and fatigue further contribute to the interplay between musculoskeletal instability and psychological outcomes. Studies demonstrate that individuals with hEDS show markedly reduced muscle endurance and strength despite normal muscle mass, suggesting intrinsic neuromuscular inefficiency (43). This weakness often combines with chronic pain to accelerate deconditioning, further exacerbating fatigue and disability (45). Fatigue is a particularly debilitating feature reported across EDS subtypes and exerts a broad impact on mental health: patients frequently describe “running on empty,” with reduced energy limiting education, work, and social participation, often leading to frustration, helplessness, and depressive symptoms (46). The constant anticipation of joint dislocation, especially in hEDS and aEDS, fosters hypervigilance and a fear of movement that can resemble trauma-related anxiety, while vEDS imposes a unique psychological burden due to the ever-present threat of catastrophic arterial rupture (47). These subtype-specific risks underscore how musculoskeletal fragility translates into psychological insecurity, reinforcing the biopsychosocial nature of EDS. Importantly, these mind–body links form bidirectional feedback loops: chronic pain and fatigue drive anxiety and depression, which in turn exacerbate muscle tension, avoidance of movement, and further deconditioning (41). Effective interventions, therefore, require integrated multidisciplinary strategies. Tailored physiotherapy to improve proprioception and muscle strength, alongside psychological interventions such as cognitive-behavioural therapy or mindfulness, has been shown to reduce pain-related anxiety and improve confidence in movement, helping to break the cycle of physical and emotional decline (38, 43). Thus, EDS exemplifies a condition in which musculoskeletal pathology is inseparable from emotional well-being, with connective tissue fragility, motor unit inefficiency, and chronic pain not only driving disability but also shaping the lived psychological experience of patients.

## 7 Linking neurological and psychiatric mechanisms in Ehlers–Danlos syndrome

**Figure 3:**
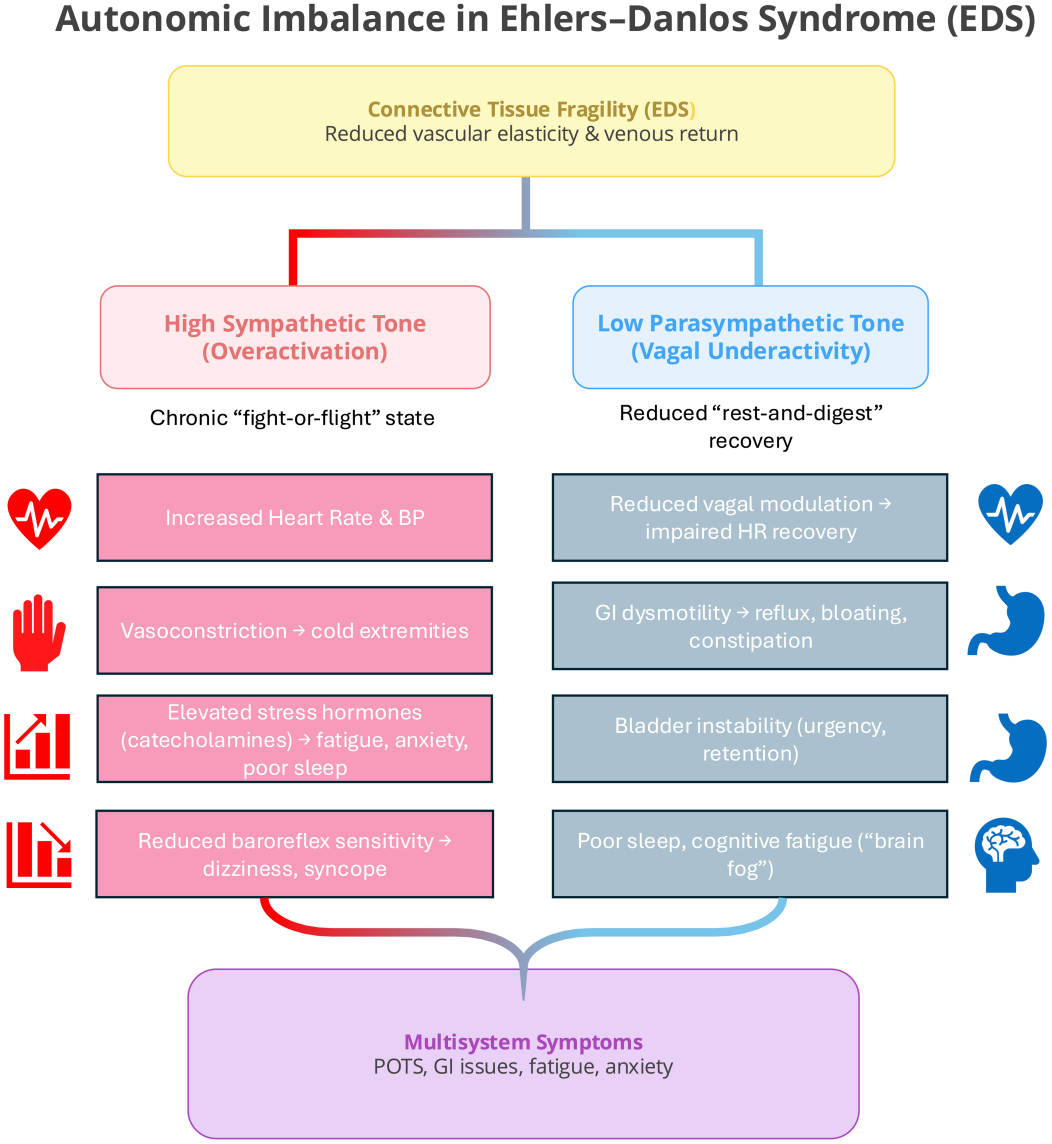
Autonomic imbalance in Ehlers–Danlos Syndrome (EDS). Connective tissue fragility leads to reduced vascular elasticity and venous return, predisposing EDS patients to chronic sympathetic overactivation and reduced parasympathetic recovery. The resulting imbalance contributes to multisystem symptoms such as POTS, fatigue, gastrointestinal dysmotility, and anxiety through sustained physiological arousal and impaired vagal modulation.

Autonomic dysregulation is one of the most consistently reported features across EDS subtypes, particularly in hypermobile EDS (hEDS), where postural orthostatic tachycardia syndrome (POTS) and related dysautonomia’s are common (21, 22). These conditions reflect sustained sympathetic overactivation with reduced parasympathetic recovery, resulting in chronic physiological arousal and heightened awareness of bodily sensations. This autonomic imbalance can contribute to anxiety, fatigue, and sleep disturbance, while increased sensitivity to interoceptive cues, such as palpitations, dizziness, or breathlessness (see Figure 3), may reinforce fear responses through feedback between visceral sensations and emotional interpretation (27). In this way, autonomic instability provides a credible biological pathway linking physiological imbalance to psychiatric vulnerability in EDS.

Neurovascular dysfunction provides another important route through which EDS may influence emotional and cognitive function. Collagen-related vascular fragility and altered cerebral perfusion have been observed in both hEDS and vEDS (28, 47), potentially resulting in intermittent cerebral hypoperfusion and associated “brain fog,” attentional lapses, and executive difficulties (20). Reduced or unstable blood flow to limbic and prefrontal regions may impair emotional regulation and increase stress sensitivity (34, 35). In addition, blood–brain barrier vulnerability and low-grade neuroinflammation (33) may further disrupt affective control through cytokine-mediated effects on neural circuits involved in emotion and cognition.

A further mechanistic link may lie in neurodevelopmental and sensory integration differences. Increasing evidence indicates higher rates of neurodivergent traits, including those associated with autism spectrum disorder (ASD) and Attention-Deficit/Hyperactivity Disorder (ADHD), among individuals with joint hypermobility and EDS (27, 44). These overlaps likely arise from shared alterations in cerebellar–thalamic–prefrontal networks that underpin proprioception, motor control, and executive functioning. Imprecise proprioceptive feedback may lead to sensory uncertainty and heightened vigilance, while from a predictive coding perspective, unreliable interoceptive and proprioceptive input could produce a persistent mismatch between expected and actual bodily states. This mismatch may underpin the hypervigilance, emotional dysregulation, and attentional instability often described in EDS (48).

## 8 Neuropsychological impact—anxiety, mood disorders, and emotional dysregulation

Emotional dysregulation refers to difficulties in managing the intensity, duration, or expression of emotions (49–51). In EDS patients, Emotional dysregulation can manifest as heightened emotional reactivity and fear, intolerance of uncertainty and difficulties in coping with stress (52). Such emotional presentations are commonly associated with anxiety disorders. While there is documented evidence that anxiety is more prevalent in individuals with EDS, much less is known about why this might occur, or the neural correlates of dysregulated emotions. The first aspect to be considered is the role of interoception; the perception of internal bodily states. Typically associated with the insula in fMRI paradigms on healthy controls (53) collagen deficits in EDS patients may disrupt visceral and vascular integrity (40), leading to aberrant interoceptive signals. Such signals may be interpreted as threats that, in turn, perpetuate the symptoms of anxiety. Furthermore, impaired prefrontal modulation of emotions (54), and heightened amygdala response may also contribute further to the emotional dysregulation that EDS patients experience (40). These findings have been further corroborated in a recent investigation (55) which found participants with hypermobility exhibited reduced neural responses to emotional faces in regions such as the inferior frontal gyrus and anterior cingulate cortex. Notably, those with both hypermobility and generalised anxiety disorder showed increased activity in the left amygdala and mid-insula, areas associated with threat processing and interoception. Furthermore, the severity of hypermobility in anxious individuals correlated with heightened anterior insula activity. These results suggest that the predisposition to anxiety in hypermobile individuals involves dynamic interactions between brain regions responsible for threat assessment and bodily state representation.

There is also evidence to suggest that an association exists between EDS and mood disorders, for example psychological studies of EDS patients have shown elevated scores on measures of alexithymia (the difficulty in identifying emotions) and hyperarousal suggesting an association between physical instability and emotional distress. Garcia Campayo et al. (56) found a high prevalence of hEDS (61.8%) among subjects suffering from panic disorders compared with 10.9% among healthy controls. Furthermore, a meta-analysis (41) revealed that individuals with hEDS experience significantly greater perception and fear intensity and have a higher probability of agoraphobia and panic disorders. Although these studies can only demonstrate correlative effects, it is evident from the literature reviewed above that an association likely exists between EDS and anxiety disorders, with emotional dysregulation potentially stemming from increased amygdala reactivity and impaired prefrontal modulation resulting in heightened fear perception, panic disorders, and depression (41, 42, 44).

It is also likely that symptoms commonly endured by EDS patients, such as chronic pain, influence emotional symptoms (36). Autonomic dysfunction and chronic pain may exacerbate neuropsychiatric symptoms (46) which may influence how interoceptive signals are received [interoceptive predictive coding; Zhou et al. (48)]. The psychological burden of EDS, including delayed diagnosis, limited awareness, and complex treatment strategies, may further exacerbate distress (57). Access to treatment, support, guilt and difficulty with sexual relationships has also been shown to further exacerbate psychological symptoms (36). A large-scale Swedish study found a higher risk of mood disorders, anorexia nervosa, obsessive-compulsive disorder, and addiction among individuals with EDS (58). The presence of obsessive-compulsive disorder and addictive behaviours in EDS may also reflect a broader tendency toward perseverative cognitive traits. Such traits, which include repetitive thought patterns and difficulty disengaging from intrusive mental content, are consistent with the heightened vigilance and perseveration reported in many EDS patients. This link suggests that perseveration may act as a shared cognitive style underlying both compulsive and addictive behaviours within this population.

More broadly, a review by Bair et al. (59) highlighted that chronic pain conditions are strongly comorbid with depression, but that this relationship is frequently mediated by anxiety and pain severity. Pasquini et al. (60) observed a higher rate of depressive symptoms in JHS/hEDS patients compared to controls; however, anxiety was not controlled for in their analyses, raising the possibility that elevated depression scores may partly reflect underlying anxiety. This points to anxiety as a potentially central mechanism in the psychiatric manifestations associated with JHS/hEDS. Other studies also revealed higher depressive symptoms in individuals with joint hypermobility without a known diagnosis of JHS/hEDS (38, 42). Similarly, Hershenfeld et al. (39) report a 42.5% prevalence of psychiatric disorders (especially depression and anxiety) in a retrospective sample of JHS/hEDS subjects which further supports the potential link. In terms of cognitive function, there is also a suggestion that EDS patients may suffer issues with attention, memory and spatial cognition (61), which may impact daily activities and increase the psychological burden of EDS patients. Bourdon et al. (60) conducted clinical evaluations of patient reports which revealed deficits such as poor concentration, memory lapses, and difficulties with orientation. The authors highlighted that contributing factors such as fatigue, joint and chronic pain, anxiety and depression can exacerbate cognitive issues, and which in turn may link to dysregulated emotions. Indeed, neuropsychological symptoms seem to exist in EDS patients irrespective of their subtype (62) so more emphasis should be placed on understanding the mind–body interaction in EDS.

**Figure 4:**
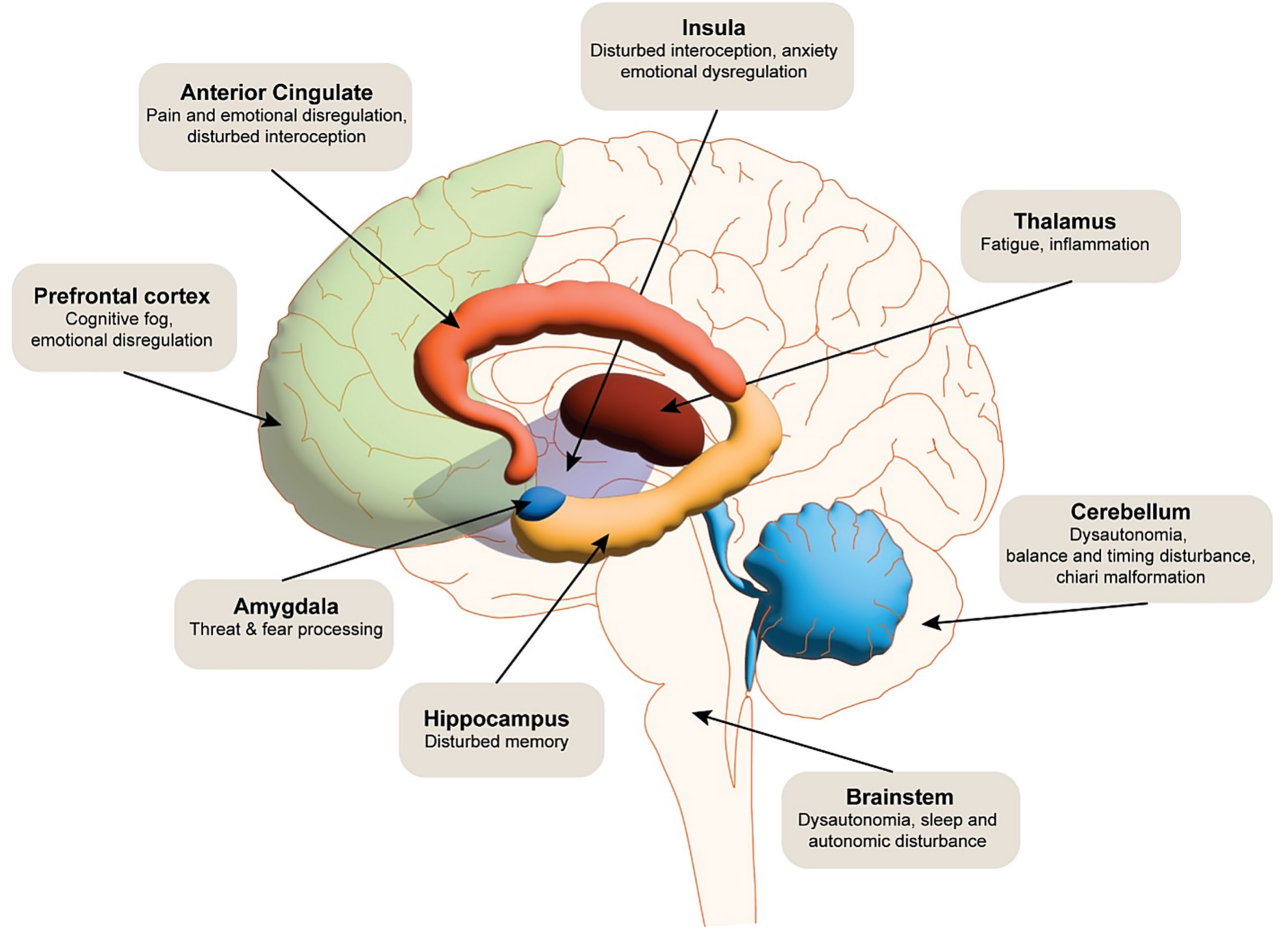
The schematic illustrates the multidimensional neural impact of EDS. Functional dysregulation is noted in cortical and subcortical regions, including the prefrontal cortex, anterior cingulate cortex, hippocampus, amygdala, insula, and somatosensory cortices, contributing to impairments in executive function, emotional regulation, interoception, and nociceptive processing. Dysautonomia-related disruptions in brainstem and hypothalamic circuits are implicated in autonomic instability frequently observed in EDS. Cerebrovascular abnormalities, such as altered cerebral perfusion and potential blood–brain barrier dysfunction, may further exacerbate neurocognitive and pain-related symptoms.

Brain regions including the anterior cingulate cortex (63–65), amygdala (66–69), insula (53, 70, 71) and prefrontal cortex (51, 69, 72–74) have all been shown to be associated with emotional dysregulation within general neuroimaging literature. In particular, amygdala hyperactivity disrupts the filtering of irrelevant stimuli, leading to an overgeneralised emotional response (e.g., heightened arousal to neutral or ambiguous cues) (51). Hallam et al. (51) noted increased amygdala activation in response to auditory stressors in conjunction with reduced inhibition from the orbitofrontal cortex, suggesting a breakdown of a more automatic process of emotional modulation. The orbitofrontal cortex (51) and the ventral medial prefrontal cortex (73–76) are thought to regulate emotions in a top-down manner, modulating activity in the amygdala. In addition to a well-documented role in interoception (see reviews (77–79)), the insula is thought to serve as a hub between the amygdala, anterior cingulate cortex and prefrontal cortex (80–83), driving the ability to regulate emotions (see Figure 4).

## 9 Health care related stressors and their impact

Beyond subtype-specific mechanisms, difficulties accessing care represent a considerable neuropsychological stressor across EDS, potentially intensifying emotional dysregulation and cognitive strain. Delayed diagnoses (often spanning years due to limited clinician awareness) alongside difficulties around treatment and scepticism from healthcare providers, can induce feelings of frustration, helplessness, and isolation, amplifying anxiety and mood instability (36, 57). This chronic stress may exacerbate HPA axis hyperactivity, further dysregulating amygdala-prefrontal circuits and heightening emotional reactivity (46, 52). EDS patients have more difficulty accessing care, as they are required to research specialists, navigate insurance, or encounter dismissal from medical professionals. All these aspects can increase mental load, which may further exacerbate attention and memory deficits already prevalent in EDS patients (83). For rarer subtypes like aEDS, where expertise is scarce, this burden may be magnified, with patients facing additional disbelief over severe yet poorly recognised symptoms like recurrent dislocations. Qualitative reports highlight how these factors can impact on psychological resilience, with access to support and strained relationships further deepening distress (36). Addressing this requires not only improved clinician education but also an increase in studies detailing and analysing how care delays shape neuropsychological outcomes, offering a pathway to mitigate this overlooked dimension of EDS’s impact on individuals suffering with the syndrome.

It is important to note, that while symptoms such as emotional dysregulation and anxiety may be present, their presence should never be used to dismiss medical complaints, especially given that anxiety may itself reflect underlying biological vulnerability rather than psychological causation. Misattributing physical symptoms to psychological causes risks delaying appropriate care. Healthcare providers should approach EDS with a comprehensive understanding that acknowledges the interconnected, but distinct, nature of its physical and neuropsychological manifestations. Indeed, healthcare providers should also work constructively with the patient to understand their lived experience. Typically, a 10-min general practitioner (GP) appointment would not be sufficient to help these patients.

## 10 Cerebrovascular complications

Vascular Ehlers-Danlos Syndrome (vEDS), caused by mutations in the COL3A1 gene, is characterised by connective tissue fragility, particularly within the vascular system. Such fragility predisposes patients to a spectrum of cerebrovascular complications, including arterial dissections, aneurysms, and other vascular malformations, which significantly contribute to morbidity and mortality. vEDS is typically characterised as the most dangerous subtype of EDS due to the impact that this has on organs such as the heart and bowel. However, such problems, whether psychological or neurological, may also extend to the brain. This section examines the nature, prevalence, and implications of these cerebrovascular events in vEDS, emphasising the psychological burden they impose.

Cerebrovascular complications in vEDS stem from the defective synthesis or structure of type 3 collagen, a critical component of arterial walls. A seminal 30-year study by Pepin et al. (47) identified vascular complications as a primary cause of death in vEDS patients, with arterial dissections and ruptures accounting for a substantial proportion of fatal outcomes. Carotid and vertebral artery dissections, cerebral aneurysms, and rarer cerebrovascular malformations, such as arteriovenous fistulas, are among the most frequently reported events (84). These complications arise due to the inherent weakness of vascular connective tissue, compounded in some cases by parental mosaicism, where a subset of germline cells harbours COL3A1 mutations (85). The unpredictable fragility of these vessels necessitates early and regular vascular assessments to mitigate catastrophic outcomes.

Despite the elevated risk of vascular anomalies, vEDS patients exhibit a paradoxically lower incidence of ischemic and hemorrhagic strokes compared to the general population (84). This observation may reflect differences in the pathophysiology of vEDS-related cerebrovascular events, which prioritise dissection and aneurysmal rupture over thrombotic or embolic mechanisms. However, when such events occur, they can lead to significant neurological deficits, including cognitive impairment and motor dysfunction, as highlighted in case studies (86).

The psychological impact of cerebrovascular risk in vEDS is also considerable. The unpredictable and life-threatening nature of these events fosters a state of chronic anxiety and hypervigilance among patients, as documented by Berglund et al. (87). This heightened emotional burden may exacerbate the perception of physical symptoms and complicate clinical management, as patients remain acutely aware of their vulnerability to sudden vascular catastrophe. Furthermore, the mismatching of interoceptive signals (see section 4) is likely to further exacerbate these symptoms. The interplay between cerebrovascular risk and emotional distress underscores the need for a holistic approach to care, integrating psychological support with vascular and neurological care.

Cerebrovascular complications in vEDS represent a critical clinical challenge, driven by collagen-related vascular fragility and manifesting as dissections, aneurysms, and malformations. While stroke incidence appears reduced, the potential for severe neurological and emotional consequences demands proactive screening and management strategies. Future research should focus on elucidating the molecular mechanisms of COL3A1 mutations and their variable penetrance, alongside developing targeted interventions to enhance vascular resilience and patient quality of life. Furthermore, patients should also receive continued psychological support and neurological monitoring to detect microbleeds.

## 11 Conclusion

EDS presents a multifaceted array of neurological and neuropsychological challenges that transcend its connective tissue origins, affecting brain structure (Chiari, CCI), peripheral nerves (SFN), emotional regulation, cognition, and vascular integrity across its 13 subtypes. These manifestations connect to physical features and experiences of chronic pain, dysautonomia and gastrointestinal issues, all of which are often invisible from the outside; however they are debilitating in impact. Addressing this complexity requires an interdisciplinary approach, integrating neurology, psychiatry, pain management, and genetics to improve diagnosis, treatment, and patient well-being.

This review highlights significant gaps in research and understanding. Mechanistic studies of BBB permeability, using CSF cytokine profiling or dynamic contrast-enhanced MRI, are critical to validate inflammation’s role in EDS’s effect on the brain. Subtype-specific neuroimaging, building on findings like reduced ACC grey matter in hEDS (29), could map neurological correlates and clarify cognitive deficits (e.g., does CCI impair prefrontal function across subtypes?). Longitudinal cohorts are needed to disentangle mood disorder causality, separating anxiety’s primary role from depression’s symptoms, and to further understand the neural underpinnings of emotional dysregulation in EDS patients. Furthermore, cerebrovascular research should explore non-vEDS subtypes and develop targeted interventions, such as collagen-stabilising drugs or advanced endovascular techniques and how this relates to insula involvement. Consequently, this may enhance interoceptive accuracy (e.g., biofeedback), mitigate inflammation (e.g., anti-cytokine agents), and support psychological resilience (e.g., cognitive-behavioural therapy). By unravelling these mind–body interactions, future work can redefine EDS as a holistic condition and hopefully will drive more informed care for EDS patients.
